# Supplementary material for: Fast online deconvolution of calcium imaging data
Source: PLoS Comput Biol. 2017 Mar 14;13(3):e1005423. doi: 10.1371/journal.pcbi.1005423 (PMC5370160; doi:10.1371/journal.pcbi.1005423)
Supplement: S1 Appendix — The supplementary material includes a naïve isotonic regression algorithm without pooling. We generalize OASIS to the case of weighted regression and provide a mathematical proof for updates according to Eqs (12–14). We further discuss how to account for elevated initial calcium fluorescence levels and provide explicit expressions of the hyperparameter updates for an AR(2) model. (PDF) [file pcbi.1005423.s001.pdf]

Fast Online Deconvolution of Calcium Imaging Data

Johannes Friedrich[1,2*], Pengcheng Zhou[1,3], Liam Paninski[1,4]

1 Department of Statistics, Grossman Center for the Statistics of Mind, and Center for Theoretical Neuroscience, Columbia University, New York, NY, USA
2 Janelia Research Campus, Ashburn, VA, USA
3 Center for the Neural Basis of Cognition and Machine Learning Department, Carnegie Mellon University, Pittsburgh, PA, USA
4 Kavli Institute for Brain Science, and NeuroTechnology Center, Columbia University, New York, NY, USA

* j.friedrich@columbia.edu

S1 Appendix. Technical appendix

Algorithm for isotonic regression without pooling

For ease of exposition Alg A shows the pseudocode of the isotonic regression algorithm used to convey the core idea. However, this naïve implementation lacks pooling, rendering it inefficient. It repeatedly updates all values $x_{t'}, ..., x_\tau$ during backtracking and calculates the updated value using Eq (7) without exploiting that part of the sum in the numerator has already been computed as an earlier result. It is thus merely $O(T^2)$, whereas introducing pools addresses both issues and yields an $O(T)$ algorithm.

Algorithm A Isotonic regression algorithm without pools (inefficient $O(T^2)$)

Require: data $\boldsymbol{y}$
initialize $\boldsymbol{x} \leftarrow \boldsymbol{y}$
for τ in $2, ..., T$ **do** ▷ move forward until end
  $t' \leftarrow \tau$
  while $t' > 1$ and $x_{t'} < x_{t'-1}$ **do** ▷ track back
    $t' \leftarrow t' - 1$
    for i in $t', ..., \tau$ **do** $x_i \leftarrow \frac{\sum_{t=t'}^{\tau} y_t}{\tau - t' + 1}$ ▷ Eq (7)
return $\boldsymbol{x}$

Weighted regression

For sake of generality we consider the case of weighted regression with weights $\boldsymbol{\theta}$.

$$\underset{\hat{\boldsymbol{c}}, \hat{\boldsymbol{s}}}{\text{minimize}} \quad \frac{1}{2} \sum_t \theta_t (\hat{c}_t - y_t)^2 + \lambda \sum_t \hat{s}_t \quad \text{subject to} \quad \hat{\boldsymbol{s}} = G\hat{\boldsymbol{c}} \geq 0 \tag{S1}$$

This generalization is not only of theoretical interest. These weights could be used to give lower weight to time points with higher variance for heteroscedastic data, for example for the Poissonian statistics of photon counts where the variance of the fluorescence increases with its mean. Further, instead of the linear relationship between

fluorescence and calcium concentration (Eq 2) we could have a nonlinear observation model

$$y_t = f(c_t) + \epsilon_t \tag{S2}$$

where the nonlinear function f can include saturation effects. This is often taken to be the Hill equation, i.e., $f(c) = \frac{ac^n}{c^n + k_d} + b$, with Hill coefficient n, dissociation constant k_d, scaling factor a and baseline b [1]. Applying Newton's algorithm to optimize for $\hat{\boldsymbol{s}}$ (or equivalently $\hat{\boldsymbol{c}}$) results for each Newton step in a weighted constrained regression problem as in Eq (S1), which can be solved efficiently with OASIS. Hence, incorporating OASIS into Newton's algorithm enables the algorithm to handle nonlinear and non-Gaussian measurements.

For an AR(1) process introducing weights changes Eq (10) to

$$\underset{c'_{t'}}{\text{minimize}} \quad \frac{1}{2} \sum_{t=0}^{\Delta t} \theta_{t+t'} (\gamma^t c'_{t'} - y_{t+t'})^2 + \sum_{t=0}^{\Delta t} \mu_{t+t'} \gamma^t c'_{t'} \tag{S3}$$

and its solution is a modification of Eq (11) by adding the weights

$$c'_{t'} = \frac{\sum_{t=0}^{\Delta t} (\theta_{t+t'} y_{t+t'} - \mu_{t+t'}) \gamma^t}{\sum_{t=0}^{\Delta t} \theta_{t+t'} \gamma^{2t}} \tag{S4}$$

We merely need to initialize each pool as $(v_t, w_t, t_t, l_t) = (y_t - \frac{\mu_t}{\theta_t}, \theta_t, t, 1)$ for each time step t and the updates according to Eqs (12-14) guarantee that Eq (S4) holds for all values $v_i = c'_{t_i}$ as we prove in the next section.

For an AR(p) process introducing weights changes Eq (30) to

$$c'_{t'} = \frac{\sum_{t=0}^{\Delta t} \left(\theta_{t+t'} \left(y_{t+t'} - \sum_{k=2}^{p} (A^t)_{1,k} c'_{t'+1-k} \right) - \mu_{t+t'} \right) (A^t)_{1,1}}{\sum_{t=0}^{\Delta t} \theta_{t+t'} (A^t)_{1,1}^2} \tag{S5}$$

and the same modified initialization holds.

Validity of updates according to equations (12-14)

Theorem 1. *The updates according to Eqs (12-14) guarantee that Eqs (11, S4) hold for all values* $v_i = c'_{t_i}$.

Proof. We proceed by induction.

Assumption: Let for the denominator and numerator of Eq (S4) hold

$$w_i = \sum_{t=0}^{l_i - 1} \theta_{t+t_i} \gamma^{2t} \tag{S6}$$

and

$$w_i v_i = \sum_{t=0}^{l_i - 1} (\theta_{t+t_i} y_{t+t_i} - \mu_{t+t_i}) \gamma^t \tag{S7}$$

Base case: Pools are initialized as $(v_t, w_t, t_t, l_t) = (y_t - \frac{\mu_t}{\theta_t}, \theta_t, t, 1)$ for each time step t such that Eqs (S6, S7) hold.

Induction step: Consider two pools (v_i, w_i, t_i, l_i) and $(v_{i+1}, w_{i+1}, t_{i+1}, l_{i+1})$ that satisfy Eqs (S6, S7) and are merged to pool (v'_i, w'_i, t'_i, l'_i) according to Eqs (12-14).

$$
\begin{aligned}
w'_i = w_i + \gamma^{2l_i} w_{i+1} &= \sum_{t=0}^{l_i-1} \theta_{t+t_i}\gamma^{2t} + \sum_{t=0}^{l_{i+1}-1} \theta_{t+t_{i+1}}\gamma^{2l_i}\gamma^{2t} \\
&= \sum_{t=0}^{l_i+l_{i+1}-1} \theta_{t+t_i}\gamma^{2t} = \sum_{t=0}^{l'_i-1} \theta_{t+t'_i}\gamma^{2t}
\end{aligned}
$$

where we used the contingency of the pools, $t_{i+1} = t_i + l_i$. Thus after the update Eq (S6) holds for the merged pool too. It remains to show this also for the values:

$$
\begin{aligned}
w'_i v'_i &= w_i v_i + \gamma^{l_i} w_{i+1} v_{i+1} \\
&= \sum_{t=0}^{l_i-1} \left(\theta_{t+t_i} y_{t+t_i} - \mu_{t+t_i}\right)\gamma^t + \sum_{t=0}^{l_{i+1}-1} \left(\theta_{t+t_{i+1}} y_{t+t_{i+1}} - \mu_{t+t_{i+1}}\right)\gamma^{l_i}\gamma^t \\
&= \sum_{t=0}^{l_i+l_{i+1}-1} \left(\theta_{t+t_i} y_{t+t_i} - \mu_{t+t_i}\right)\gamma^t = \sum_{t=0}^{l'_i-1} \left(\theta_{t+t'_i} y_{t+t'_i} - \mu_{t+t'_i}\right)\gamma^t
\end{aligned}
$$

□

Initial calcium fluorescence

Thus far we have not explicitly taken account of elevated initial calcium fluorescence levels due to previous spiking activity. For the case $p = 1$ positive fluorescence values c_1 capture initial calcium fluorescence that decays exponentially. Positive values c_1 lead via $\boldsymbol{s} = \boldsymbol{G}\boldsymbol{c}$ to a positive spike s_1. Instead of attributing the elevated fluorescence to a spike at time $t = 1$, a positive s_1 more likely accounts for previous spiking activity. Therefore we remove the initial spike by setting $s_1 = 0$ (Alg 2, line 12).

For $p = 2$ we can model the effect of prior spiking activity as an exponential decay, too. Because the validity of the constraint $c_t \geq \sum_{i=1}^{p} \gamma_i c_{t-i}$ can only be evaluated if $t > p$, for $p > 1$ the first pool stays thus far merely at its initialization $(y_1 - \mu_1, y_1 - \mu_1, 1, 1)$, and the noisy raw data value is taken as true c_1. Instead, we suggest to use the first pool to model the exponential decay due to previous spiking activity. Given $c_1 = v_1$ the fluorescence values c_t for $t = 1, ..., l_1$ are then given by $d^{t-1}c_1$ with decay variable

$$
d = \tfrac{1}{2}(\gamma_1 + \sqrt{\gamma_1^2 + 4\gamma_2}) \tag{S8}
$$

as well known in the AR / linear systems literature [2]. The first pool is merged with the second one whenever the constraint $v_2 \geq d^{l_1} v_1$ is violated.

Explicit expressions of the hyperparameter updates for AR(2)

We solve the noise constrained problem by increasing λ in the dual formulation until the noise constraint is tight. We start with some small λ, e.g. $\lambda = 0$, to obtain a first partitioning into pools $\mathcal{P}$.

We denote all except the differing last two components of $\boldsymbol{\mu}$ by $\mu = \lambda(1 - \gamma_1 - \gamma_2)$ (Eq 27) and express the components of $\boldsymbol{\mu}$ as $\mu_t = \mu\,\kappa_t$ with

$$
\kappa_t = \begin{cases} \frac{1}{1-\gamma_1-\gamma_2} & \text{if} \quad t = T \\ \frac{1-\gamma_1}{1-\gamma_1-\gamma_2} & \text{if} \quad t = T-1 \\ 1 & \text{else.} \end{cases} \tag{S9}
$$

Given some $\mu(\lambda)$, the value of the first pool used to model the initial calcium fluorescence is (using Eq 11)

$$\hat{c}_1 = \frac{\sum_{t=1}^{l_1}(y_t - \mu\,\kappa_t)d^{t-1}}{\sum_{t=0}^{l_1-1} d^{2t}} \tag{S10}$$

with decay factor d defined in Eq (S8). The other values in this first pool are implicitly defined by

$$\hat{c}_t = d\,\hat{c}_{t-1} \quad \text{for} \quad t = 2, ..., l_1. \tag{S11}$$

The values of the other pools are according to Eq (30)

$$\hat{c}_{t_i} = \frac{\sum_{t=0}^{l_i-1}\left(y_{t_i+t} - \mu\,\kappa_{t_i+t} - (A^t)_{1,2}\hat{c}_{t_i-1}\right)(A^t)_{1,1}}{\sum_{t=0}^{l_i-1}(A^t)_{1,1}^2} \tag{S12}$$

The other values in the pool are implicitly defined by

$$\hat{c}_{t_i+t} = \gamma_1\hat{c}_{t_i+t-1} + \gamma_2\hat{c}_{t_i+t-2} \quad \text{for} \quad t = 1, ..., l_i - 1. \tag{S13}$$

Altogether these equations define $\hat{\boldsymbol{c}}(\mu)$ as function of μ. The solution $\hat{\boldsymbol{c}}' = \hat{\boldsymbol{c}}(\mu')$ for an updated value $\mu' = \mu + \Delta\mu$ is linear in $\Delta\mu$

$$\hat{\boldsymbol{c}}' = \hat{\boldsymbol{c}} - \Delta\mu\boldsymbol{f} \tag{S14}$$

which plugged in above Eqs (S10-S13) yields that $\boldsymbol{f}$ can be evaluated using the following equations by plugging in the numerical values of γ_1, γ_2, d, $\boldsymbol{\kappa}$, A and $\{l_i\}$

$$f_1 = \frac{\sum_{t=1}^{l_1}\kappa_t d^{t-1}}{\sum_{t=0}^{l_1-1} d^{2t}} \tag{S15}$$

$$f_t = d\,f_{t-1} \qquad \text{for} \quad t = 2, ..., l_1 \tag{S16}$$

$$f_{t_i} = \frac{\sum_{t=0}^{l_i-1}\left(\kappa_{t_i+t} - (A^t)_{1,2}f_{t_i-1}\right)(A^t)_{1,1}}{\sum_{t=0}^{l_i-1}(A^t)_{1,1}^2} \qquad \text{for} \quad i = 2, ..., z \tag{S17}$$

$$f_{t_i+t} = \gamma_1 f_{t_i+t-1} + \gamma_2 f_{t_i+t-2} \qquad \text{for} \quad t = 1, ..., l_i - 1 \tag{S18}$$

where z is the index of the last pool and because pools are updated independently we make the approximation that no changes in the pool structure occur. Inserting Eq (S14) into the noise constraint (Eq 15) and denoting the residual as $\boldsymbol{r} = \hat{\boldsymbol{c}} - \boldsymbol{y}$ results in

$$\|\hat{\boldsymbol{c}}' - \boldsymbol{y}\|^2 = \|\hat{\boldsymbol{c}} - \Delta\mu\boldsymbol{f} - \boldsymbol{y}\|^2 = \|\boldsymbol{r} - \Delta\mu\boldsymbol{f}\|^2 = \|\boldsymbol{f}\|^2\Delta\mu^2 - 2\boldsymbol{r}^\top\boldsymbol{f}\Delta\mu + \|\boldsymbol{r}\|^2 \stackrel{!}{=} \hat{\sigma}^2 T \tag{S19}$$

and solving the quadratic equation for $\Delta\mu$ yields

$$\Delta\mu = \frac{\boldsymbol{r}^\top\boldsymbol{f} + \sqrt{(\boldsymbol{r}^\top\boldsymbol{f})^2 - \|\boldsymbol{f}\|^2(\|\boldsymbol{r}\|^2 - \hat{\sigma}^2 T)}}{\|\boldsymbol{f}\|^2}. \tag{S20}$$

If we jointly want to optimize the baseline too, we denote again the total shift applied to the data (except for the last two time steps) due to baseline and sparsity penalty as $\phi = b + \mu$. We increase ϕ until the noise constraint is tight. The optimal baseline $\hat{b}$ minimizes the objective (20) with respect to it, yielding $\hat{b} = \langle \boldsymbol{y} - \hat{\boldsymbol{c}}\rangle = \frac{1}{T}\sum_{t=1}^{T}(y_t - \hat{c}_t)$. Appropriately adding $\hat{b}$ to the noise constraint yields

$$\|\hat{b}'\boldsymbol{1} + \hat{\boldsymbol{c}}' - \boldsymbol{y}\|^2 = \|\langle \boldsymbol{y} - \hat{\boldsymbol{c}} + \Delta\phi\boldsymbol{f}\rangle\boldsymbol{1} + \hat{\boldsymbol{c}} - \Delta\phi\boldsymbol{f} - \boldsymbol{y}\|^2 \tag{S21}$$

$$= \|\underbrace{\hat{b}\boldsymbol{1} + \hat{\boldsymbol{c}} - \boldsymbol{y}}_{\boldsymbol{r}} - \Delta\phi(\underbrace{\boldsymbol{f} - \langle\boldsymbol{f}\rangle\boldsymbol{1}}_{\bar{\boldsymbol{f}}})\|^2 \stackrel{!}{=} \hat{\sigma}^2 T \tag{S22}$$

where we used Eq (S14), the current value of the baseline $\hat{b} = \langle \boldsymbol{y} - \hat{\boldsymbol{c}} \rangle$ and the updated value $\hat{b}' = \langle \boldsymbol{y} - \hat{\boldsymbol{c}} + \Delta\phi \boldsymbol{f} \rangle$. Solving the quadratic equation for $\Delta\phi$ yields

$$\Delta\phi = \frac{(\boldsymbol{r}^\top \bar{\boldsymbol{f}} + \sqrt{(\boldsymbol{r}^\top \bar{\boldsymbol{f}})^2 - \|\bar{\boldsymbol{f}}\|^2 (\|\boldsymbol{r}\|^2 - \hat{\sigma}^2 T)}}{\|\bar{\boldsymbol{f}}\|^2}. \tag{S23}$$

References

1. Pologruto TA, Yasuda R, Svoboda K. Monitoring neural activity and [Ca^{2+}] with genetically encoded Ca^{2+} indicators. J Neurosci. 2004;24(43):9572–9579.

2. Brockwell PJ, Davis RA. Time series: theory and methods. Springer Science & Business Media, 2013.
